# The medial approach open reduction for developmental dysplasia of the hip: do the long-term outcomes validate this approach? A systematic review of the literature

**DOI:** 10.1007/s11832-014-0612-1

**Published:** 2014-09-30

**Authors:** Oluwasegun Akilapa

**Affiliations:** Kings College Hospital, London, England

**Keywords:** Medial approach open reduction, Developmental dysplasia of the hip, Systematic review

## Abstract

**Introduction:**

Developmental dysplasia of the hip (DDH) is one of the most commonly diagnosed and treated paediatric orthopaedic conditions.

**Objective:**

To systematically identify, appraise and synthesise the best evidence for the long-term outcomes of the medial approach open reduction (MAOR) for DDH.

**Methods:**

MEDLINE, EMBASE and the Cochrane databases were searched up to July 2013. All study designs that reported on the long-term outcomes of the MAOR as the primary treatment modality for DDH were included. The risk of bias in each study was evaluated using the Cochrane risk of bias assessment tool with some modification to accommodate different study designs.

**Results:**

From the 162 citations screened, five retrospective observational studies that fulfilled the eligibility criteria were included. The mean age at surgery varied from 10 to 17 months with an average follow-up period of 16–25 years. Acetabular development, as defined by the Severin Classification, was reported as satisfactory (Severin I/II) in between 38 and 79 % of study cohorts. However these good and excellent outcomes were less promising when patients who had additional operations were considered as unsatisfactory results. Avascular necrosis, as predominantly defined by the Kalamchi criteria, varied from 5 to 43 %. Negative prognostic factors implicated were mean age at surgery >17 months, the absence of the ossific nucleus and eccentric posturing of the femoral head postoperatively. The rate of secondary operations reported varied from 11 to 50 %. There were no reported total hip replacements.

**Conclusion:**

There is a paucity of robust evidence pertaining to the long-term outcomes of the MAOR for developmental dysplasia of the hip. The trends from observational studies suggest that the long-term outcomes are not as positive as short- to intermediate-term studies suggest. Further prospective, controlled and rigorously designed studies are required to validate this approach.

## Introduction

The medial approach to the hip was first described over a 100 years ago. The pioneer surgeon, Ludloff, described an approach to the hip via an anteromedial incision that facilitated access to the principal structures perceived as responsible for hip instability [1, 2]. Over the past few decades, other luminaries in the field of paediatric orthopaedics have validated the medial approach, albeit with some modifications of their own [3, 4].

Conflicting reports of the success and failures of the approach from short- [4, 5] and intermediate-term [6] studies question the validity of this approach especially when considering the long-term consequences of persistent acetabular dysplasia, e.g. the need for major hip replacement surgery as a young adult.

There are very few studies that have explored the outcomes of the medial approach open reduction (MAOR) up to or beyond skeletal maturity [7–9]. In keeping with the theme of uncertainty, some of these reports have suggested that the long-term outcomes of the medial approach are dubious [7, 8], while others have attempted to validate it as the panacea for open reduction in developmental dysplasia of the hip [9].

In a bid to seek clarity, a systematic review addressing the long-term outcomes of the medial approach was conducted. The aim of this review was to identify, appraise and synthesise the best evidence pertaining to the long-term outcomes of the MAOR for developmental dysplasia of the hip in children.

## Methods

### Criteria for considering studies for this review

#### Types of studies

All study designs were included with the exception of single case reports, commentaries, technical notes and expert opinions.

#### Types of participants

Studies included had to report on patients who had undergone the MAOR for developmental dysplasia of the hip as the primary operative intervention. Studies were excluded if the patients had additional femoral or pelvic osteotomies simultaneously with the open reduction.

#### Types of interventions

All medial approaches to open reduction for DDH, e.g. Ludloff [1], Ferguson [3], Weinstein and Ponseti [4], etc.

#### Types of outcome measures

- Hip function as assessed by a validated patient or clinician reported outcome measure, e.g. the Harris Hip Score [10], Oxford Hip Scores [11], Hip Disability and Osteoarthritis Outcome Score [12], McKay’s criteria [13], etc.
- Avascular necrosis as assessed by a validated outcome measure, e.g. the Kalamchi criteria [14] or Salter’s criteria [15].
- Femoral head and acetabular development as assessed by validated measures, e.g. the Severin Classification [16].
- Secondary operations in the early-, intermediate- or long-term as a consequence of persistent dysplasia associated with or without hip instability, e.g. femoral or pelvic osteotomies, hip resurfacing or hip replacement surgery.

All outcomes had to be assessed at a minimum of at least 15 years of follow-up.

### Search strategy for identification of studies

The following electronic databases were searched for relevant studies:

- Ovid MEDLINE(R) <1946 to July week 5 2013>.
- EMBASE <1974 to 2013 week 32>.
- Cochrane Central Register of Controlled Trials (CENTRAL)

The search strategies, completed on the 24 July 2013, have been documented in Appendices 1 and 2. Studies were limited to those published in the English language. In addition to the databases searched, conference proceedings for the British Society for Children’s Orthopaedic Surgery (BSCOS), the European Paediatric Orthopaedic Society (EPOS) and Pediatric Orthopaedic Society of North America (POSNA) over the last 5 years were also searched for relevant articles. A manual search of cited references from retrieved articles was also done to increase the sensitivity of the electronic search strategy.

### Study selection

The titles and abstracts of all articles retrieved via the combined electronic and manual search strategy were reviewed against the pre-determined eligibility criteria. Full texts of relevant articles were retrieved to identify the studies with eligible patient cohorts, the appropriate surgical intervention and follow-up for at least 15 years.

### Data collection

Data were extracted from the included studies using a modified version of the Strengthening the Reporting of Observational Studies in Epidemiology (STROBE) checklist [17]. The information extracted relates to the study design and duration, participant number and demographics, specific interventions, outcome and time points collected and reported and a detailed analysis of the results.

### Assessment of methodological quality

The methodological quality of included studies was assessed using a modification of the Cochrane Collaboration’s tool for assessing risk of bias [18]. The risk of the various sources of bias— selection, performance, detection, attrition and reporting—was assessed against the background of the observational design of the included studies. Summary judgements were made within the context of the strengths and weaknesses of the study types included.

### Data synthesis

The included studies displayed too much heterogeneity to justify a meta-analysis. As a consequence the evidence extracted was summarised in a narrative synthesis.

## Results

**Fig. 1:**
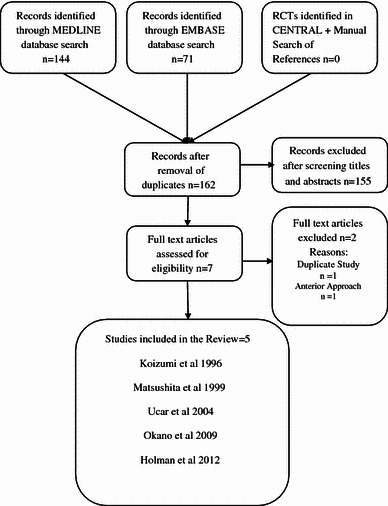
Study flow diagram

The combined electronic and manual search strategies retrieved 162 articles. A preliminary review of all titles and abstracts against the pre-determined eligibility criteria led to an initial exclusion of 155 articles. A review of the full text of the remaining seven articles led to a further two exclusions. One article was a duplicate [19] of one of the included studies [20] and the other article focused on the anterior as opposed to MAOR [21]. At the end of the screening process, five studies were included in this review [7–9, 20, 22]. The flow chart below (Fig. 1) shows the methodological transition from the initial set of identified records to those finally included in the review.

### Included studies

#### Holman et al. (2012)

Holman and colleagues [9] conducted a retrospective review of 53 patients (66 hips) treated by either the Ludloff MAOR (18 hips) or anterior open reduction (48 hips) for developmental dysplasia of the hip between 1955 and 1995 across four specialist children hospitals in the USA. The mean age at surgery for the medial approach group was 1.4 years (0.41–3.5 years) with an average follow-up time of 25 years.

The eligibility criteria, though explicitly reported, raised some questions about the integrity of the selection process. An unspecified number of participants was excluded on basis of postoperative radiological criteria considered as poor open reductions. Exclusion of projected failures may have further compounded the risk of a systematic selection bias inherent in the retrospective study design.

The internal and external validity of the intervention (MAOR) was difficult to assess as the authors did not provide adequate details of several facets of the intervention (preoperative interventions, precise surgical technique and breadth of surgical expertise, postoperative immobilisation, etc.). Although blinding was not feasible in the operative facet of the interventions, the lack of reporting of precise indications for allocation to either treatment group erodes confidence about attempts to minimise performance bias. Furthermore, there is an unclear risk of detection bias as authors did not provide any details of attempts to blind the outcome assessors, which was feasible for some outcomes in spite of the retrospective study design.

The reported outcomes (functional scores, early re-dislocations, Severin classification, avascular necrosis, secondary operations) were relevant and assessed with validated outcome measures. The risk of selective reporting bias was adjudged as low. Fifty-six per cent (10/18 hips) of patients treated via the medial approach had good or excellent outcomes (Severin I/II) at an average of 25 years post primary surgery. Half of the hips (9/18) required further surgery for residual dysplasia. Avascular necrosis was reported in a single patient (5.5 %).

Eighteen out of the 38 hips originally treated by the medial approach were included in the follow-up study. The proportion of patients (53 %) lost to follow-up rendered the study vulnerable to attrition bias.

The authors concluded that the results of open reduction for DDH deteriorate as the age at surgery increases and identified redislocation and avascular necrosis as poor prognostic indicators. Caution has to be applied in the interpretation of the true impact of these prognostic factors as the deductions were made from a combined analysis of patients in both the medial and anterior approach groups. Overall, the study offers a rare glimpse into the long-term outcomes of the MAOR for DDH 25 years postoperatively. The limited confidence in the rigor of the selection process undermines the study’s ability to compare the relative efficacy of the medial and anterior approach.

#### Okano et al. (2009)

Okano and colleagues [8] conducted a retrospective review of 43 patients (45 hips) from an original cohort of 49 patients treated by the Ludloff MAOR for DDH between 1979 and 1997 in a single centre in Japan. The mean age at surgery (MAOR) was 14 months (6–31 months) and the average follow-up period was 16.4 years (10–28 years). The authors provided limited information about the selection process, e.g. inclusion and exclusion criteria over the 18-year recruitment period; hence it was not possible to make clear judgements about the internal or external validity of the study selection process. The risk of selection bias was adjudged as high.

The pertinent facets of the surgical intervention (preoperative treatment, operative technique, postoperative immobilisation) were explicitly reported. However, the patients did not receive standardised interventions as intraoperative judgements led to preferential partial excision of the labrum in about a third of the included cohort. Furthermore five patients had additional operations (Shelf with derotation varus osteotomy, Chiari pelvic osteotomy and a rotational acetabular osteotomy) between 3 and 16 years postoperatively. The risk of performance bias was adjudged as high.

The outcomes reported were explicit and assessed with validated measures. In addition to the six patients lost to follow-up, the authors reported further exclusions of three patients who had additional operations before the age of 10 and another two patients who had an osteotomy after 10 years postoperatively from the clinical evaluation. These exclusions would have undermined the validity of the clinical outcomes. Confidence in the validity of radiological measurements was strengthened by the reported coefficient of variation as well as independent assessment of the relevant outcomes.

The overall incidence of avascular necrosis (Kalamchi) was 29 % and poor acetabular development (i.e. Severin III upwards) was reported in 60 % of the entire cohort. The authors implicated age at surgery over 17 months as a bad prognostic factor for both outcomes. The risk of selective reporting bias for both clinical radiological outcomes was adjudged as low. The follow-up rate of 88 % was high, especially when considering radiological outcomes. However the exclusions prior to the analysis of clinical outcomes may have introduced a systematic attrition bias.

Overall, the study’s strength revolves around attempts to minimise detection bias (radiological outcomes) and its relatively high follow-up rate. Its weaknesses with regards to the selection process and inconsistencies in some facets of the intervention warrant caution in the interpretation of the results.

#### Ucar et al. (2004)

Ucar and colleagues [20] performed an evaluation of the Ferguson’s MAOR in a group of 30 patients (44 hips) with an average age at initial surgery of 10.7 (2–19 months) and a mean follow-up of 19.8 years (13–27.5 years). The included participants were part of a historic cohort of 37 patients (56 hips) treated by a single surgeon between 1974 and 1989 at an orthopaedic centre in Turkey. The reporting of the selection process in the current [20] or historic publication [23] did not convey sufficient confidence about the external validity of the study population.

Relevant facets of the surgical intervention (preoperative treatment, operative technique, postoperative immobilisation) performed by a single surgeon were explicitly reported. The surgical technique was not standardised across all patients as certain components (adductor tenotomy and ligamentum teres excision) of the procedure were dependent on intraoperative judgements about their relative contributions to instability. Furthermore, additional surgery was necessary in 11 hips (25 %), thus compromising the homogeneity of the intervention within the study cohort. The risk of performance bias was rated as high.

The reported outcomes were relevant and assessed with validated outcome measures. The authors reported excellent clinical outcomes (modified McKay criteria and Iowa Hip Rating) in all but one patient. Acetabular development (Severin Classification) was reported as excellent or good in 79 % of hips and clinically relevant avascular necrosis (Kalamchi criteria type 2 upwards) was detected in eight hips (18 %) at skeletal maturity. Patients who had additional operations for residual dysplasia were significantly older than patients who did not require additional surgery (mean age 15.7 months vs. 9.6 months, *p* = 0.001). The incidence of AVN was significantly lower in the presence of the ossific nucleus of the femoral head at initial surgery than its absence (*p* = 0.033). The outcomes were clearly defined although there was no evidence to suggest that the outcome assessors where blinded to minimise detection bias.

Seven patients (12 hips) were lost to follow-up. This represents an attrition of about 20 %, which inspires a little bit of confidence bearing in mind the longevity of the follow-up period. The risk of attrition bias was adjudged as low.

#### Matsushita et al. (1999)

Matsushita and colleagues [22] compared the long-term clinical and radiological outcomes of the Ludloff open reduction and a wide exposure method (360-degree circumferential capsulotomy) in a combined cohort of 51 patients (63 hips) treated across two specialist children hospitals in Japan between 1973 and 1980. Twenty-seven patients (32 hips) were treated by the medial approach at a mean age at surgery of 12 months (5–30 months) with an average follow-up period of 16 years (11–20 years). Children treated by the wide exposure (WE) method were a bit older (mean age 18 months, range 12–31 months) and had a similarly lengthy follow-up. The enrolment process was not explicitly documented. Treatment allocation was stratified by hospital without any documented criteria or evidence of attempts to conceal the interventions. The risk of selection bias was considered high.

The interventions were explicitly documented. Apart from the surgical approaches, distinct differences were reported in the postoperative posture in hip spica (30° WE vs. 70° MAOR of abduction) as well as the duration of immobilisation (2 months WE vs. 6 months MAOR). Furthermore, 11 out of 32 hips (34.4 %) in the medial group had additional operations compared to none in the wide exposure group. As allocations to treatment were not systematically protected, the overall risk of performance bias was also adjudged as high.

There was no significant difference between groups on clinical grounds (modified McKay criteria). However the authors reported significantly better radiological outcomes (Severin criteria) in the wide exposure group compared to the medial approach (*p* < 0.05). Fifty-six per cent of the hips treated via the medial approach had satisfactory outcomes (Severin I/II) compared to 83.9 % in the wide exposure group. Avascular necrosis, as assessed by Salter’s criteria, was reported in a single hip (3.2 %) in the wide exposure group compared to seven hips (21.9 %) in the medial group. The authors proposed that the medial approach was technically inadequate because of its inability to address the tension in the posterosuperior part of the capsule and short external rotators, predisposing to subluxation of the femoral head.

There was no documented attempt to blind the outcome assessors; hence the risk of detection bias was adjudged as unclear. There was also insufficient information about the overall eligible cohort, selection criteria and recall rate. The risk of attrition bias was judged as unclear.

#### Koizumi et al. (1996)

Koizumi and colleagues reviewed 33 patients (35 hips) after open reduction of developmental dysplasia of the hip using the Ludloff approach 20 years postoperatively. The included participants were from a cohort of 51 patients (55 hips) treated at a specialist children’s hospital in Japan. The mean age at surgery was 14 months (5–29) and mean age at follow-up was 20.1 years (15–24). The authors reported explicit eligibility criteria but did not provide adequate details about the enrolment sequence, recruitment time frame, etc., raising questions about the external validity of the selection process. The risk of selection bias was rated as high.

The relevant facets of the surgical intervention were reported in explicit and easily reproducible detail. However, the authors made a significant modification of the surgical technique (no psoas tenotomy) with no attempt to validate the omission. A hypertrophied psoas tendon has been implicated as a major constraint to a concentric reduction [1] and its omission may have had significant ramifications on the success or failure of the procedure. In addition to the primary open reduction, 16 out of 35 hips (46 %) were operated again because of persistent dysplasia during the intervening study period. Overall, the risk of a performance bias was considered as high.

The authors did not report on any patient-reported outcome measure but assessed relevant radiological outcomes with validated measures. Acetabular development (Severin) was reported as poor in a majority of patients (54.3 %). Avascular necrosis (Kalamchi type II upwards) was also reported as remarkably high (42.9 %). The risk of selective reporting bias for radiological outcomes was rated as low but it was challenging to make a judgement as to whether patient-reported outcomes were not assessed at all or systematically omitted in the report.

The authors reported that approximately 35 % of hips were lost to follow-up. The authors did not make any statistical assumptions (conservative or otherwise) about the outcomes of the hips (or patients) lost to follow-up and presented the results using the responsive cohort as the baseline. Risk of attrition bias was adjudged as high.

A summary of the study characteristics and risk of bias judgements is highlighted in Tables 1 and 2.

**Table 1:** Descriptive summary of included studies

Study	Participants	Intervention(s)	Comparator	Outcomes
Holman et al. [9] Follow-up: 25 years Retrospective series Recruitment: 1955–1995 Multicentre, US	18 hips Eligible MAOR cohort: 38 hips Mean age at surgery: 1.4 years (Range 5–41 months) Mean follow-up(MAOR): 25 years (range 14–35 years)	Ludloff MAOR, limited details provided No specific documentation of structures released or details of postoperative immobilisation protocols	Anterior open reductions 48 hips	Follow-up rate (MAOR): 47 % Severin I/II MAOR; 10/18Hips (56 %) AVN: 1/18 (5.5 %) Additional operations MAOR: 9/18 hips (50 %) Varus Derotation osteotomies X 2 Pemberton osteotomies X 6 Periacetabular osteotomy
Okano et al. [8] Follow-up: 16.4 years Retrospective series Recruitment: 1979–1997 Japan	43 patients (45 hips) Eligible cohort: 49 patients Mean age at surgery: 14 months (range 6– 31 months) Mean follow-up: 16.4 years (range 10–28 years)	Preoperative Pavlik harness or Failed closed reductions Ludloff MAOR Anterior capsulotomy, Ligamentum teres excision, Psoas tenotomy, TAL excision, Partial labrum excision:13 patients Hip spica × 4 weeks (90’ flexion, 70’ abduction) Abduction brace 4–6 months	None	Follow-up rate: 88 % Severin I/II: 18/45 hips (40 %) AVN (Kalamchi): 13/45 hips 29 % McKay criteria: 92.3 % satisfactory Additional operations: 5/45 Shelf, Chiari pelvic osteotomy Rotational Acetabular osteotomy
Ucar et al. [20] Follow-up: 19.8 years Retrospective series Single surgeon	30 patients (44 hips) Eligible cohort: 37 patients 1974–1989, Turkey Mean age at surgery: 10.7 months (range 2–19 months) Mean follow-up: 19.8 (range 13–27.5 years)	Ferguson MAOR, inverted T-capsulotomy, Psoas tenotomy, TAL excision Optional adductor tenotomy Optional LT excision Hip spica: human position ×3/12 Abduction brace: 6 months	None	Follow-up rate: 78 % McKay criteria: 96 % satisfactory Severin I/II: 35/44 hips (79 %) AVN (Kalamchi): 9/44 hips (20 %) Additional operations: 11/44 hips 25 % Salters (7), femoral derotation osteotomy (4), femoral varus osteotomy (2)
Matsuishita 1999 [22] Follow-up: 16 years Retrospective series Single surgeon 1973–1980, Japan	27 patients (32 hips) MAOR Mean age at surgery: 12 months (range 5–30 months) Mean follow-up: 16 years	Preoperative: Pavlik harness, traction, closed reduction Ludloff MAOR Adductor tenotomy, Psoas tenotomy Anteroinferior capsulotomy Ligementum teres excision Optional labrum excision Hip spica × 4/52 (90° flexion, 70° abduction) Harness/abduction brace × 6 months	Wide exposure *Circumferential capsulotomy *Transection of hip abductors + short external rotators *Psoas tendon transfer, LT + TAL excision	No significant difference between groups on clinical grounds (McKay criteria) Severin I/II WE: 83.9 % Severin I/II MAOR; 56.3 % AVN WE (Salter’s): 3.2 % AVN MAOR (Salter’s): 21.9 % Additional operations WE: nil Additional operations MAOR: 34 %
Koizumi et al. 1996 [7] Follow-up: 19.4 years Retrospective series	33 patients (35 hips) from a cohort of 51 patients in Japan Mean age at surgery: 14 months (range 5–30 months) Mean follow-up: 19.4 years (range 14–23 years)	Modified Ludloff MAOR Anterior capsulotomy Ligamentum teres excision TAL excision No psoas tenotomy Hip spica × 4 weeks (90° flexion, 90° abduction) Abduction brace × 6 months	None	Follow-up rate: 65 % Severin I/II: 16/35 hips (45 %) AVN (Kalamchi): 15/35 hips (42.9 %) Additional operations: 16/35 hips (46 %) Salter’s osteotomy X 6, Pemberton X 5 Pemberton + DVO x 2, Chiari(1) Shelf + DVO(1) Rotational acetabular osteotomy + DVO (1)

*MAOR* medial approach open reduction, *AVN* avascular necrosis, *WE* wide-exposure, *TAL* tranvsverse acetabular ligament, *LT* ligamentum teres, *DVO* derotational varus osteotomies

**Table 2:**
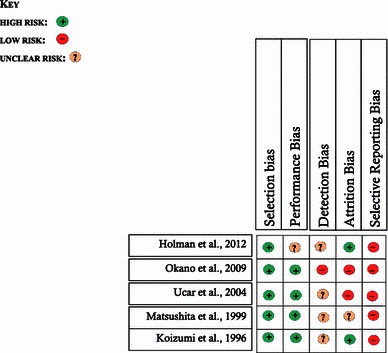
Summary of risk of bias of the included studies

## Discussion

The objective of this review was to identify, appraise and synthesise the best evidence pertaining to the long-term outcomes (up to and beyond skeletal maturity) of the MAOR for developmental dysplasia of the hip in children. No randomised, quasi-randomised or prospective study was identified in the literature. Five retrospective, observational studies that met the inclusion criteria have been critically appraised and summarised with particular emphasis on the most relevant long-term outcomes.

Four of the included studies [7, 8, 20, 22] had a combined cohort of 133 patients (158 hips). The fifth study involved 18 hips but the number of patients were not reported [9]. The mean age at surgery spanned between 10 and 16 months with a mean follow-up period from 16 to 25 years.

Pre-MAOR interventions included treatment with the Pavlik harness, overhead traction or closed reduction stabilised with a hip spica. The Ludloff medial approach to the hip was the standard surgical technique across all but one study [20], which favoured Ferguson’s approach. There were several inconsistencies within studies that reportedly adopted the same Ludloff approach. Koizumi and colleagues did not perform a psoas tenotomy [7], others performed conditional excision of the adductor tendon, ligamentum teres [20] limbus [8, 22], etc., based on intraoperative judgments about prerequisites for a concentric reduction. There were also notable variations in the posturing of the patients among those with hip spica. The position varied from 90 to 110 degrees of flexion and 45 to 90° of abduction.

Two studies made direct comparisons between the medial and other surgical approaches. Holman et al. [9] compared their cohort with another group of patients that had an open reduction via the anterior approach. Matsushita and colleagues [22] attempted to validate a wide exposure method (circumferential capsulotomy) by comparisons with their medial group. The retrospective nature of both studies did not make it feasible to demonstrate a robust selection process capable of addressing the distribution of known and unknown confounders between treatment groups.

The answer to the pertinent question “Do the long-term outcomes validate this approach?” has been addressed under the following subheadings; avascular necrosis, acetabular development, additional operations and hip function.

### Avascular necrosis

Avascular necrosis (AVN) of the femoral head was graded according to the Kalamchi and MacEwen’s classification [24] in three of the included studies [7, 8, 20], Salter’s criteria [15] in a single study [22] and undefined criteria in the final study [9]. The median reported rate was 21.9 % (range 5–43 %), which lies well within the 0–67 % jurisdiction reported in the literature [3, 14, 25–31]. Its external validity is undermined by the heterogeneity in various facets of the interventions, inconsistencies in outcome measures and variable attrition rates. Prognostic factors highlighted across these long-term studies include age at surgery [8], perioperative microvascular insult [20], a tight posterior capsule [22] and eccentric posturing of the femoral head in plaster [7]. The study that reported the highest rate (43 %) of avascular necrosis attributed their poor results to a technical deficiency of the medial approach [7]. The authors suggest that AVN was a consequence of eccentric reduction postoperatively. It is interesting to note that the patients in their cohort were immobilised in 90° of abduction. Bucholz et al. [32] postulated that abduction in this position could lead to compression of the medial circumflex vessels between the labrum of the acetabulum and the neck of the femur. Bache and colleagues [6] reported higher AVN rates when the postoperative abduction in spica (measured by MRI) was greater than 60°. Gardner et al. [33] also implicated abduction of more than 60° in spica as a risk factor for avascular necrosis.

Investigators in the included studies also implicated age at surgery as a prognostic factor for avascular necrosis. However the correlations defined were not consistent enough to validate an optimal age range that rendered immunity to the development of this phenomena. Okano and colleagues [8] reported poor prognosis in patients older than 17 months as at the time of primary open reduction. Ucar et al. noted older age at surgery was protective against AVN although this did not achieve statistical significance. In the wider literature, authors have suggested that the Ludloff approach is a safe and effective method for the treatment of congenital dislocation of the hip in infants who are less than 24 months of age [25, 34]. However conflicting data from a univariate analysis reported a higher rate of avascular necrosis when surgery was performed in children aged <12 months [33].

Ucar and colleagues [20] also suggested that the ossific nucleus is protective against avascular necrosis as its occurence was significantly lower in the presence of the ossific nucleus of the femoral head than in its absence (*p* = 0.033). Bache et al. [6] postulated the opposite and reported a significant relationship between AVN and the absence of the ossific nucleus.

The incidence of avascular necrosis (within included studies) appeared to increase with the length of follow-up. Ucar et al. [20] highlighted the fact that AVN rates more than doubled (from 8.9 to 20 %) in the same cohort of patients assessed at 8 and 24 years postoperatively. Koizumi and colleagues implicated Kalamchi type II necrosis for deterioration after 10 years of age in hips that became unacceptable radiologically. In the wider literature, studies with intermediate-term [27, 34] follow-up have reported higher AVN rates than studies with shorter term follow-up [5, 28].

## Acetabular development

The Severin Classification was utilised across all the included studies [7–9, 20, 22] to assess long-term acetabular development. Although its use for DDH has been validated by a widely cited study [35], the interobserver reliability in particular, has been questioned in more recent studies [36, 37]. Within this review, study-specific percentages of Severin I/II outcomes varied from less than 50 [7, 8] to within 50 and 60 [9, 22] to approximately 80 % [20]. The outcomes were even less positive when patients who had additional operations were also classified as unacceptable.

Three of the included studies indicated the mean age at surgery [8, 9, 20] as a prognostic factor for acetabular development. Okano and colleagues [8] suggested that open reduction after 17 months of age was a bad prognostic factor. Holman et al. [9] reported significantly lower ages at surgery between patients in Severin groups I, II, III compared with Severin IV with a propensity towards poor results over the age of 3. Ucar [20] reported that patients who underwent additional surgery for residual dysplasia were significantly older than patients who did not require surgery. In the wider literature, studies with shorter term follow-up (prior to skeletal maturity) have reported that patients operated on before the age of 2 years have good results [6, 25, 26, 30, 38]. Although it is challenging to define an exact age cutoff, it appears that early intervention bodes well for satisfactory acetabular development at skeletal maturity.

Koizumi et al. [7] as well as Matsushita [22] questioned the technical adequacy of the medial approach. Both authors suggested that the surgeons' inability to address extra-articular impediments such as a tight posterosuperior capsule and contracted short external rotators led to eccentric reductions, poor femoral-acetabular congruity and consequently poor acetabular development. It is noteworthy to mention that Koizumi and colleagues did not perform a psoas tenotomy in any of their patients, although it has been established as a major extra-articular obastacle to a concentric reduction [5, 6, 39].

The efficacy of the medial approach open reduction, in its own right, is further confounded by additional operations performed before skeletal maturity. A conservative estimate of the successes attributable to this procedure alone that considers additional operations as failures of acetabular development (Severin III upwards) erodes confidence about the efficacy of the medial approach. The reported rates of successful outcomes (Severin I/II) at skeletal maturity for patients who had the MAOR exclusively were 23 % [7], 34.4 % [22], 40 % [8] and 59 % [20]. The results suggest that the medial approach as a solitary procedure does not guarantee successful hip maturation.

## Additional operations

The rate of secondary operations reported ranged from 11 [8] to 50 % [9] across all included studies. The number of additional operations reported in historical series varied from 25 to 65 % [25–27, 40]. Operations included Pemberton, Salter’s, Chiari’s and shelf osteotomies, etc. There was no reported total hip arthroplasty in any study, bearing in mind the oldest patient in the combined cohorts was 37 years of age.

The spectrum of indications included redislocation, residual subluxation, residual dysplasia, deformed femoral heads and osteoarthritis. The prognostic factors were consistent with those identified for poor acetabular development or the development of avascular necrosis. Older patients at surgery were more likely to have additional operations [8, 9, 20]. Bache and colleagues reported that children who had reductions after the age of 12 months required secondary operations 70 % of the time and were three times more likely to require secondary procedures when compared to younger infants [6].

Patients who had a medial as opposed to wide circumferential capsulotomy were also reported as more likely to need secondary operations [22]. This was in constrast to reports in another comparative cohort in which 15 % of patients required total hip arthroplasties in the anterior group compared to none in the medial group [9]. In the wider literature, there is no clear distinction between these approaches without additional surgery and as such a prospective randomised study comparing exclusive results at skeletal maturity is long overdue.

## Hip function

Three studies [8, 20, 22] reported the patient’s functional status with the Modified McKay criteria [13] with ratings in the good or excellent range between 91 and 97 % of patients. Holman and colleagues [9] utilised the Harris Hip and WOMAC scores but did not provide exclusive estimates for the anterior or medial approach groups. A single study did not report on patient functional status at all [7]. The clinical outcomes definitely portrayed a more optimistic outlook than the radiological outcomes, which is likely to translate to deferred interventions such as major joint replacement surgery.

## Implications for future research

All five studies included in this review are retrospective, observational studies. As highlighted in the assessment of the risk of bias previously discussed, the cumulative methodological rigour was compromised by a high risk of selection and performance bias. Furthermore the lack of blinding of outcome assessors coupled with the significantly high attrition rates introduces further risk of detection and attrition bias. Overall, further prospective, controlled, rigorously conducted studies are likely to impact on the level of confidence on the estimates of the outcomes assessed in this review and may change the reported trends (outcomes of the medial approach worsen with the length of follow-up).

The heterogeneity of the included studies made it challenging to collectively quantify the impact of any reported prognostic factor independently. Relatively younger age at surgery for example was suggested as protective with regards to acetabular growth and development [8, 9, 20] as well as destructive with regards to avascular necrosis [20]. Hence the impact of age or the exact prognostic cutoff varied in different studies as well as with the outcomes assessed.

In conclusion, this review sought to identify, appraise and synthesise the best evidence pertaining to the long-term outcomes (up to and beyond skeletal maturity) of the MAOR for developmental dysplasia of the hip in children. The research question posed—“Do the long-term outcomes validate its use for developmental dysplasia of the hip?”—cannot be answered unequivocally based on the strength of evidence available in the current literature. The potential impact of this review on clinical practice relates to its more measured depiction of the successes and failures of the medial approach well beyond skeletal maturity. It can serve as an invaluable part of the decision-making processes right from the first consultation in the paediatric orthopaedic clinic preoperatively through to specialist hip care, if necessary, decades later.
